# Supplementary material for: Study on the Changes in Immobilized Petroleum–Degrading Bacteria Beads in a Continuous Bioreactor Related to Physicochemical Performance, Degradation Ability, and Microbial Community
Source: Int J Environ Res Public Health. 2022 Sep 9;19(18):11348. doi: 10.3390/ijerph191811348 (PMC9517540; doi:10.3390/ijerph191811348)
Supplement: Supplementary file 1 [file ijerph-19-11348-s001.zip › ijerph-1870783-supplementary.pdf]

Table Caption

Table S1. Composition of mineral salt medium (MSM)

Table S2. Abundance in functional classification of samples

Figure Caption

Figure S1. Design of bioreactor and preparation on immobilized petroleum degrading bacteria beads.

Table S1. Composition of mineral salt medium (MSM)

Chemicals	Usage amount
NaCl	30 g L^{-1}
KH_2PO_4	0.5 g L^{-1}
Yeast powder	2 g L^{-1}
Na_2HPO_4	0.6 g L^{-1}
$(NH_4)_2SO_4$	5 g L^{-1}
$CaCl_2 \cdot FeSO_4 \cdot MgSO_4$	1 mL L^{-1}

Table S2. Abundance in functional classification of samples

Category	OS	IB	EB	Description
A	16257	3897	1386	RNA processing and modification
B	27871	8202	3584	Chromatin structure and dynamics
C	2873975	2153606	2669282	Energy production and conversion
D	462718	280207	314327	Cell cycle control, cell division, chromosome partitioning
E	4125068	3081202	4000944	Amino acid transport and metabolism
F	1126135	824392	1080498	Nucleotide transport and metabolism
G	2627791	2045401	2943310	Carbohydrate transport and metabolism
H	1786562	1267216	1587493	Coenzyme transport and metabolism
I	1753912	1365463	1697583	Lipid transport and metabolism
J	2588862	2044678	2702569	Translation, ribosomal structure and biogenesis
K	2177726	1772671	2215129	Transcription
L	2215100	1500005	1806874	Replication, recombination and repair
M	2873921	2453052	3357644	Cell wall/membrane/envelope biogenesis

N	470507	288105	178713	Cell motility
O	1881799	1370423	1637493	Posttranslational modification, protein turnover, chaperones
P	2315398	2147226	2793774	Inorganic ion transport and metabolism
Q	997443	736226	921473	Secondary metabolites biosynthesis, transport and catabolism
R	3486953	2987638	4137255	General function prediction only
S	3477255	2959514	3805493	Function unknown
T	2374590	1721245	2000468	Signal transduction mechanisms
U	755871	544138	551425	Intracellular trafficking, secretion, and vesicular transport
V	651520	579775	839269	Defense mechanisms
W	67	36	8	Extracellular structures
Z	9609	827	285	Cytoskeleton

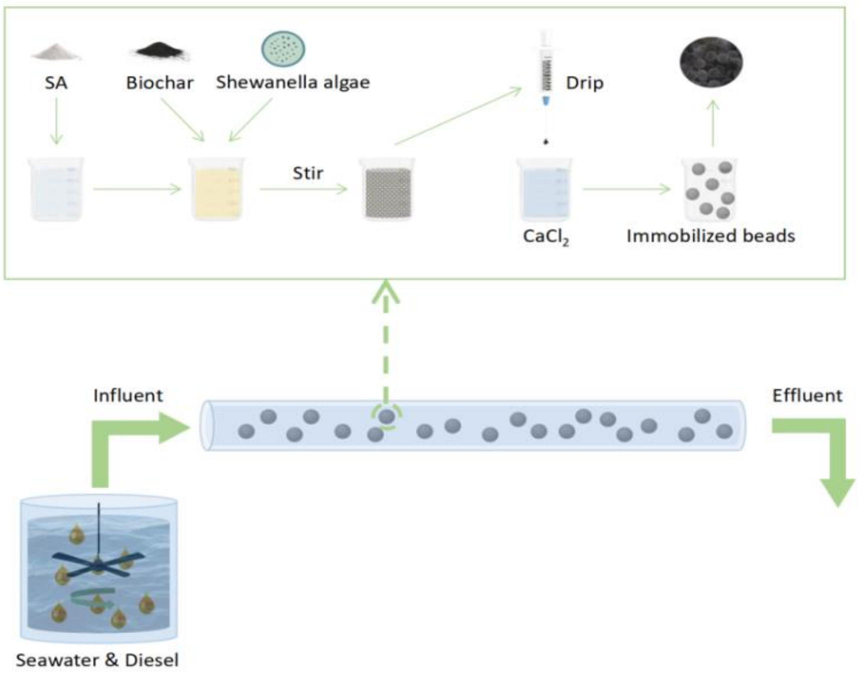

Figure S1. Design of bioreactor and preparation on immobilized petroleum degrading bacteria beads.
